# Supplementary material for: Pre-Pregnancy Weight and Symptoms of Attention Deficit Hyperactivity Disorder and Executive Functioning Behaviors in Preschool Children
Source: Int J Environ Res Public Health. 2019 Feb 25;16(4):667. doi: 10.3390/ijerph16040667 (PMC6406951; doi:10.3390/ijerph16040667)
Supplement: Supplementary file 1 [file ijerph-16-00667-s001.zip › Supplementary Table S1.pdf]

Supplementary Table S1. Association between pre-pregnancy BMI, gestational weight gain, and childhood BASC ADHD symptoms among children born at 37 weeks gestation or greater (n=296).

**Model**	**ADHD**				**Hyperactivity**				**Attention Problem**			
	B	*se*	*ß*	*p*	*B*	*se*	*ß*	*p*	*B*	*se*	*ß*	*p*
**Model 1**												
BMI (continuous)	**0.17**	**0.05**	**0.21**	**0.002**	**0.10**	**0.04**	**0.17**	**0.02**	**0.07**	**0.02**	**0.20**	**0.001**
**Model 2**												
BMI (categorical)												
0 - 24.9 (referent)												
25 - 29.9	0.10	1.14	0.01	0.93	-0.36	0.90	-0.03	0.69	0.51	0.44	0.06	0.24
30 - 34.9	-0.07	1.03	-0.002	0.95	-0.16	0.72	-0.01	0.83	0.10	0.58	0.01	0.87
> 35	**4.43**	**1.40**	**0.22**	**0.002**	**2.62**	**1.10**	**0.19**	**0.02**	**1.80**	**0.53**	**0.20**	**0.001**
**Model 3**												
Gestational weight gain (kg) (continuous)	-0.004	0.06	-0.004	0.95	-0.03	0.04	-0.05	0.47	0.03	0.03	0.06	0.34
**Model 4**												
Gestational weight gain (categorical)												
Less than adequate	**2.89**	**1.33**	**0.16**	**0.03**	**2.10**	**0.95**	**0.16**	**0.03**	0.80	0.61	0.10	0.19
Adequate (referent)												
More than adequate	1.12	0.93	0.08	0.23	0.45	0.65	0.04	0.49	0.67	0.39	0.10	0.09

B: unstandardized regression coefficient; **se:** robust standard error; **ß:** standardized coefficient; **p:** p-value; Models 1 and 2 adjusted for gestational diabetes, parity, birth weight, child's age, sex, mother's age, race, education, gestational weeks, smoking and mother's concurrent ADHD; Models 3 and 4 adjusted for pre-pregnancy BMI, parity, birth weight, child's age, sex, mom's age, race, education, gestational weeks, smoking and mother's ADHD.
