# Supplementary material for: Pre-Pregnancy Weight and Symptoms of Attention Deficit Hyperactivity Disorder and Executive Functioning Behaviors in Preschool Children
Source: Int J Environ Res Public Health. 2019 Feb 25;16(4):667. doi: 10.3390/ijerph16040667 (PMC6406951; doi:10.3390/ijerph16040667)
Supplement: Supplementary file 1 [file ijerph-16-00667-s001.zip › Supplementary Table S2.pdf]

**Supplementary Table S2. Association between pre-pregnancy BMI, gestational weight gain, and childhood BRIEF GEC score among children born at 37 weeks gestation or greater (n=296).**

| **Model** | **GEC score** | | | | **Inhibit** | | | | **Shift** | | | | **Emotional Control** | | | | **Working Memory** | | | | **Plan/Organize** | | | |
|---|---|---|---|---|---|---|---|---|---|---|---|---|---|---|---|---|---|---|---|---|---|---|---|---|
| | *B* | *se* | *ß* | *p* | *B* | *se* | *ß* | *p* | *B* | *se* | *ß* | *p* | *B* | *se* | *ß* | *p* | *B* | *se* | *ß* | *p* | *B* | *se* | *ß* | *p* |
| **Model 1** | | | | | | | | | | | | | | | | | | | | | | | | |
| BMI (continuous) | **0.40** | **0.17** | **0.17** | **0.02** | **0.10** | **0.05** | **0.14** | **0.04** | **0.06** | **0.03** | **0.15** | **0.04** | 0.04 | 0.03 | 0.09 | 0.20 | **0.12** | **0.05** | **0.18** | **0.02** | **0.07** | **0.03** | **0.18** | **0.004** |
| **Model 2** | | | | | | | | | | | | | | | | | | | | | | | | |
| BMI (categorical) | | | | | | | | | | | | | | | | | | | | | | | | |
| 0 - 24.9 (referent) | | | | | | | | | | | | | | | | | | | | | | | | |
| 25 - 29.9 | 0.38 | 3.05 | 0.01 | 0.90 | 0.01 | 1.01 | 0.001 | 0.99 | 0.26 | 0.46 | 0.03 | 0.56 | -0.39 | 0.53 | -0.04 | 0.46 | 0.44 | 1.01 | 0.03 | 0.67 | -0.07 | 0.54 | -0.01 | 0.90 |
| 30 - 34.9 | -0.99 | 3.74 | -0.01 | 0.79 | -0.69 | 1.08 | -0.03 | 0.53 | -0.68 | 0.64 | -0.06 | 0.29 | -0.57 | 0.91 | -0.04 | 0.53 | 0.43 | 1.05 | 0.02 | 0.68 | 0.40 | 0.66 | 0.03 | 0.54 |
| > 35 | **9.81** | **4.45** | **0.17** | **0.03** | 2.43 | 1.19 | 0.14 | 0.04 | 1.36 | 0.72 | 0.14 | 0.06 | 1.13 | 0.80 | 0.10 | 0.16 | **3.31** | **1.40** | **0.20** | **0.02** | **1.58** | **0.71** | **0.16** | **0.03** |
| **Model 3** | | | | | | | | | | | | | | | | | | | | | | | | |
| Gestational weight gain (kg) (continuous) | 0.20 | 0.15 | 0.08 | 0.18 | 0.04 | 0.05 | 0.05 | 0.39 | 0.02 | 0.02 | 0.05 | 0.38 | 0.02 | 0.03 | 0.04 | 0.56 | 0.06 | 0.04 | 0.08 | 0.16 | **0.05** | **0.03** | **0.11** | **0.04** |
| **Model 4** | | | | | | | | | | | | | | | | | | | | | | | | |
| Gestational weight gain (kg) (categorical) | | | | | | | | | | | | | | | | | | | | | | | | |
| Less than adequate | 2.13 | 4.07 | 0.04 | 0.60 | 1.04 | 1.13 | 0.07 | 0.36 | 0.40 | 0.65 | 0.05 | 0.54 | 0.81 | 0.77 | 0.08 | 0.29 | -0.004 | 1.21 | -0.00 | 1.00 | -0.62 | 0.70 | -0.07 | 0.38 |
| Adequate (referent) | | | | | | | | | | | | | | | | | | | | | | | | |
| More than adequate | 2.49 | 3.08 | 0.06 | 0.42 | 0.75 | 0.85 | 0.06 | 0.38 | 0.40 | 0.49 | 0.06 | 0.41 | 0.33 | 0.57 | 0.04 | 0.57 | 0.30 | 0.91 | 0.02 | 0.74 | 0.14 | 0.52 | 0.02 | 0.79 |

B: unstandardized regression coefficient; **se:** robust standard error; **ß:** standardized coefficient; **p:** p-value; Models 1 and 2 adjusted for gestational diabetes, parity, birth weight, child's age, sex, mother's age, race, education, gestational weeks, smoking and mother's concurrent ADHD; Models 3 and 4 adjusted for pre-pregnancy BMI, parity, birth weight, child's age, sex, mom's age, race, education, gestational weeks, smoking and mother's ADHD.
